# Development of a Dog-Assisted Activity Program in an Elementary Classroom

**DOI:** 10.3390/vetsci4040062

**Published:** 2017-11-27

**Authors:** Cinzia Correale, Lara Crescimbene, Marta Borgi, Francesca Cirulli

**Affiliations:** 1 Mind Association, 00161 Rome, Italy; cinziacorreale@gmail.com; 2 K-9 Italy srl, 00138 Rome, Italy; lara.crescimbene@gmail.com; 3 Center for Behavioral Sciences and Mental Health, Istituto Superiore di Sanità, 00161 Rome, Italy; francesca.cirulli@iss.it

**Keywords:** animal-assisted intervention, children, classroom pets

## Abstract

Here we describe a pilot Dog-Assisted Activity program that was designed to improve wellbeing and social integration in a multi-cultural elementary classroom in which some episodes of bullying had been reported. We developed a 5-encounters protocol with the aim of introducing pet dogs into the class to stimulate understanding of different types of communication and behavior, ultimately facilitating positive relationships among peers. A preliminary evaluation was carried out in order to assess the effect of the program on teachers’ perception of children’s difficulties (e.g., peer relationship problems) and strengths (prosocial behaviors) by means of a brief behavioral screening tool, the Strengths and Difficulties Questionnaire (SDQ—Teacher version). Overall results indicate that, by means of the recognition of the dogs’ behavior and non-verbal communication, children were able to express their emotions and to show behaviors that had not been recognized by the teachers prior to the intervention. In particular, the SDQ Total Difficulties scores suggest that the teacher had increased awareness of the students’ difficulties as a result of the dog-assisted program. Overall, the presence of animals in the educational environment may provide enjoyment and hands-on educational experiences, enhanced psychological wellbeing, and increased empathy and socio-emotional development.

## 1. Introduction

Schools and other educational settings represent an important environment for children’s social and emotional development; in these contexts, it is indeed of particular importance to promote a calming atmosphere and to stimulate the development of skills such as managing emotions and relationships with peers and adults, being focused and following directions [1].

According to the Biophilia hypothesis [2], friendly animals are able to have a calming effect on humans, particularly on children, and to increase alertness and attention, ultimately enhancing concentration and task persistence. Moreover, animals are thought to be able to promote relationships among peers, by encouraging approach to others and engagement in social interactions [3].

Based on these assumptions, over the last decade, Animal-Assisted Interventions have become increasingly popular in educational settings [4,5,6]. Bringing small animals into the classroom, dogs visiting the classroom with a handler, and bringing a dog owned by the teacher to the school on a regular basis are some examples of these activities [7,8,9]. The main aims of educational interventions assisted by pets are to improve students’ attention and discipline, to promote student-teacher relationships, to prompt creative activities, to teach humane attitudes and responsibility for a living being, and to motivate students with learning problems and other difficulties [5,7,9,10,11,12,13,14,15]. Dogs are the most popular pet, possibly because of their ability to act as a “social lubricant”, and to provide a calm atmosphere, as well as their value as rewards and motivators [9,16,17]. 

Here, we describe a pilot Dog-Assisted Activity program that was designed to improve wellbeing and social integration in a multi-cultural elementary classroom in which some episodes of bullying had been reported. The intervention was prompted by a specific request from the special educational needs teacher who was attempting to find strategies for positive classroom management. In particular, the teacher reported some difficulties in managing behavioral problems and stress manifestations in children, and in identifying students who were more in need of support, as well as in focusing on students’ strengths, in order to create an inclusive climate and promote social integration into the classroom.

To respond to the teacher’s requests, we developed a 5-encounters protocol with the aim of enhancing the observation, knowledge and understanding of the value of what is “different” (i.e., of a different species) in children, to enhance children’s understanding of different types of communication, and to facilitate positive relationships among peers through the mediation of the dog. A preliminary evaluation of the intervention was carried out. In particular, here we propose the use of a brief behavioral screening (SDQ) that covers a range of attributes including children’s difficulties (e.g., peer relationship problems) and strengths (prosocial behavior), as observed by the teacher.

## 2. Methods

The intervention took place in a public elementary school located in Rome, Italy. Twenty-one children (12 males, 9 females) between 9 and 10 years old were involved. One child had a diagnosis of Specific Learning Disorder. The intervention consisted in 5 biweekly encounters. Each encounter lasted 1 h and a half. The team in charge of the activity was composed by 2 dogs (1 English setter called “Brenno” and 1 Miniature pinscher called “Otello”), one dog handler, one educator and a psychologist for ecological observations and supervision. All team members received a three-level formal education according to the “National Guidelines for Animal Assisted Interventions” of the Italian Ministry of Health. These guidelines were drawn to deliver specific rules concerning the roles (and the training) of the animal handler, as well as of the teacher/educator, and give very clear indications as to how to monitor animal welfare. No Animal Ethics approval was needed, since no procedure (defined as “pain caused by the insertion of a needle”) was performed on the animals, in compliance with European and relative National regulations for animal experimentation (Directive 2010/63/EU; Legislat. Decree no. 26, 4 March 2014). With regard to human ethical approval, according to the Italian Legislation, ethical consent is needed only for clinical trials (Legislat. Decree no. 211 of 24 June 2003, Transposition of Directive 2001/20/EC relating to the implementation of good clinical practice in the conduct of clinical trials on medicinal products for clinical use). The Istituto Superiore di Sanità is covered by an assurance of compliance (Human Subject Assurance Number: FWA0000413); a commitment to maintain policies and procedures for protecting the rights and welfare of human participants. Parents gave their informed consent for their children to participate to the program.

Before starting the intervention, some information about the two dogs (including some pictures) was provided to the children in order to help familiarize them with the animals. Moreover, participants were asked to fill a questionnaire in order to collect information about their familiarity with dogs, ownership, and their attitudes towards dogs. Activities proposed to students are shown in Table 1. 

During the encounters, the educator actively engaged children in the activities, working individually or in groups. The dog handler helped children to observe dogs and to understand and interpret their behavior, as well as to interact properly with them (e.g., petting, approaching them). She was also in charge of monitoring animal welfare during the encounters and to promptly intervene in case the dogs manifested signs of stress (e.g., excessive manifestation of calm-signs, avoiding/redirecting behaviors, etc.) by interrupting the session (in case of persistent signs of stress) or by changing activity, keeping distance between the dog and the stressing stimulus. Teachers were always present and ready to respond to any request by children; they observed the encounters “from the outside”, and were not actively involved in the lesson. This allowed them to better observe all children; in particular, how they behaved with the dogs and the team members, and how they interacted with the other students. They were focusing on students’ strengths and difficulties emerging during the encounters. 

Before the first (T0) and after the last (T1) encounter, the Strengths and Difficulties Questionnaire (SDQ—Teacher version) [18,19] was administered to the special educational needs teacher. The SDQ is a brief behavioral screening questionnaire about 3–16-year-old children. It covers a range of attributes, in particular, emotional symptoms, conduct problems, hyperactivity/inattention, peer relationship problems (all these sub-items being summarized in a total difficulties score) and pro-social behavior. The supplemental “Impact” score gives information on distress, social impairment, and burden to others. The SDQ uses multiple rater sources; there exist parent, teacher, and self-report versions. In this program, the teacher version of the SDQ was used. Additional data were collected by the psychologist using the research method of “paper and pencil” diary entries, which allows the noting of subtle aspects related to emotional behavior and interactions among peers by means of ecological observations of the classroom. 

## 3. Results

Scores from the SDQ (Teacher version) are reported in Table 2. Only the child with the diagnosis of Specific Learning Disorder had a borderline Total Difficulties score and abnormal Conduct Problems, Hyperactivity/Inattention and Impact scores. One child had a borderline Peer Problems score, another received an abnormal Hyperactivity/Inattention score, with a borderline Impact score. None of the children was rated as having difficulties in the Prosocial behavior domain. At the end of the program (T1), SDQ was administered to the same special educational needs teacher. As for children rated as having difficulties at T0, at T1 they all received increased (abnormal) Total Difficulties scores. The special educational needs teacher was interviewed (unstructured interview) at T1, after the administration of the SDQ. During the interview, she reported that, observing the activities “from the outside” (and not being actively involved in the lesson as usual)—in addition to having the presence of the dogs acting as “social mediator”—allowed her to better understand children’s behavior (towards the dogs, the team members and the other students) and to more accurately recognize their difficulties. These results are consistent with the observations made by the psychologist, who used the research method of the “pen and pencil” diary to monitor children’s behavior during interactions with peers and dogs. 

## 4. Discussion

Here, we have described a pilot dog-assisted educational program aimed at promoting social integration in a multi-cultural elementary classroom. The protocol proposed was developed with the aim of introducing pet dogs into the class to stimulate understanding of different types of communication and behavior, ultimately facilitating positive relationships among peers through the mediation of the dogs. The study was carried out in order to assess the effect of the program on teachers’ perception of children’s difficulties (e.g., peer relationship problems) and strengths (prosocial behaviors) by means of a brief behavioral screening (SDQ).

Although it is difficult to draw firm conclusions, given the pilot nature of the study and the lack of a control group, overall results indicate that, by means of the recognition of dog’s behavior and their non-verbal communication, children were able to express their emotions and to show behaviors that had not been recognized by the teachers prior to the intervention. In particular, the SDQ Total Difficulties scores increased between T0 and T1, a result that suggests the teacher’s increased awareness of students’ difficulties as a result of the dog-assisted program. The teacher’s observations, also expressed during the informal interview, were consistent with those of the psychologist (annotated in the diary). It is of interest that, although the request was prompted by the special education teacher, who was in charge of a student with some difficulties, this brief program was able to allow the detection of some difficulties in other children that had been overlooked prior the intervention.

These observations may represent a first step that might encourage the application of dog-assisted programs to target specific behavioral/relational problems and to promote social integration in educational contexts, informing feasibility in the design of larger studies and suggesting assessment tools, such as the SDQ. The use of the SDQ questionnaire appears to have allowed teachers to become more aware of some behavioral problems that had been previously underestimated, as well as specific strengths characterizing some of the children.

Through their presence and interaction with children, dogs have the potential to promote the establishment of a trusting student-teacher relationship, to positively affect social behavior and motivation, and to reduce aggression episodes and behavioral problems. As shown in previous research, the presence of animals in the educational environment may provide enjoyment and hands-on educational experiences, enhanced psychological wellbeing, and increased empathy and socio-emotional development [5,20]. More research is warranted in order to understand which activities are more effective, to correlate outcomes of the interventions to children’s familiarity with—and attitudes towards—animals [21], and to explore the mechanisms underlying the positive effects observed [22], particularly in relation to physiological stress reactions of students in school settings [17,23,24]. Attentional aspects of children’s relationships with pet animals should also be encouraged [25], especially in the light of recent evidence on the effect of dogs in improving cognitive learning and specific school-related tasks (e.g., reading) [26,27].

## 5. Conclusions

The preliminary evaluations carried out in this program indicate that the methodological protocol here proposed is able to advise teachers on which individuals are more in need of support. We can speculate that, following the activities with the dogs, the strengths and difficulties of some students may reveal themselves more clearly, and could subsequently be operated on by teachers. This pilot study can thus inform future (controlled) studies to be carried out in educational settings. 

## Figures and Tables

**Table 1:** Activities proposed during the dog-assisted program.

Encounter	Goal	Activities	Results
**1st encounter**: “Evolution: from wolf to dog”	Observation, knowledge and understanding of the value of what is "different”.	Group session: watching movies and images on the topic of evolution of the wolf and the domestication of the dog.	Children acquired further knowledge with regard to the natural evolution of living beings and dog domestication, including the development of a bond with human beings.
**2nd encounter**: “Exploring the world through the sense of smell”	Putting oneself in someone's shoes, experience a different point of view.	Playing with dogs: blindfolded searching activities by using the sense of smell.	Children experimented a different way to explore the world, mainly using the sense of smell instead of vision, in order to understand the psychological meaning of “putting oneself in someone's shoes”.
**3rd encounter**: “A thousand ways to communicate”	Use of nonverbal communication, understanding the importance of emotions in communication.	Team work: observation of dog behaviors and their categorization (emotional categories: happiness, sadness, insecurity, etc).	Children, divided into 4 different groups (teams), explored the range of emotions that others can feel, through the recognition of animal behavior and non-verbal communication (ears lowered, tail wagging, etc.). This experience helped them to talk about their own emotions and feelings through the observation of dog behaviors.
**4th encounter**: “How to play”	Use the dog as a mediator of the relationship with peers to promote a positive interaction.	Team work: creation of fairy tales with dogs as main characters (narrative about a conflict to be solved).	Children, divided into 4 different groups (teams), wrote and painted 8 stories in which problem/conflict they were aware of was solved: they experimented and reinforced the ideal of forgiveness and friendship, at the same time improving their narrative abilities, creativity and visual skills.
**5th encounter**: “Please, take care of me!”	Through the dog, children can experience their own interpretation of the parental role and what “taking care of others” means.	Team work: drawing T-shirts to be given as a goodbye gift to the dogs “Brenno” and “Otello”.	Children experienced their ability to “take care of someone”, by preparing and personalizing a gift for the dogs named “Brenno” and “Otello” during the last encounter. T-shirts were prepared as a symbol of their “friendship”. T-shirt were decorated with messages of love and appreciation for all the things children have had the opportunity to learn thanks to the dogs.

**Table 2:** SDQ (Teacher version) Scores.

Child	T0							T1						
	ES	CP	H	PP	PB	I	TOT	ES	CP	H	PP	I	PB	TOT
**1**	0	0	2	0	7	0	**2**	0	0	2	0	0	7	**2**
**2**	0	0	2	0	10	0	**2**	0	0	2	0	0	10	**2**
**3**	0	0	0	0	9	0	**0**	0	0	0	0	0	9	**0**
**4**	0	0	0	0	10	0	**0**	0	0	0	0	0	10	**0**
**5**	1	0	0	0	10	0	**1**	1	0	0	0	0	10	**1**
**6**	0	2	2	0	10	0	**4**	0	2	2	0	0	10	**4**
**7**	0	0	0	0	8	0	**0**	0	0	0	0	0	8	**0**
**8**	2	4 ^ǂ^	7 ^ǂ^	0	6	3 ^ǂ^	13 *	4	4 ^ǂ^	8 ^ǂ^	0	3 ^ǂ^	8	**16** ^ǂ^
**9**	0	0	0	0	10	0	**0**	0	0	0	0	0	10	**0**
**10**	0	0	0	0	10	0	**0**	0	0	0	0	0	10	**0**
**11**	0	0	0	0	10	0	**0**	0	0	0	0	0	10	**0**
**12**	0	1	2	0	10	0	**3**	0	1	2	0	0	10	**3**
**13**	2	0	1	0	10	0	**3**	4	0	1	0	0	10	**5**
**14**	2	2	2	4 *	7	0	**10**	4	5 ^ǂ^	4	5 ^ǂ^	0	7	**18** ^ǂ^
**15**	0	0	2	0	10	0	**2**	0	0	2	0	0	10	**2**
**16**	0	0	3	0	9	0	**3**	0	0	3	0	0	9	**3**
**17**	0	1	1	0	10	0	**2**	0	1	1	0	0	10	**2**
**18**	1	0	0	0	10	0	**1**	1	0	0	0	0	10	**1**
**19**	0	1	8 ^ǂ^	1	6	1 *	**10**	1	1	8 ^ǂ^	6 ^ǂ^	0	7	**16** ^ǂ^
**20**	0	0	0	0	10	0	**0**	0	0	0	0	0	10	**0**
**21**	0	1	4	0	10	0	**5**	0	1	4	0	0	10	**5**

**ES:** Emotional Symptoms Score; **CP:** Conduct Problems Score; **H:** Hyperactivity/Inattention Score; **PP:** Peer Problems Score; **PB:** Prosocial Behavior Score; **I:** Impact Score; **Total:** Total Difficulties Score. * Borderline scores; ^ǂ^ Abnormal scores.
